# A Comparative Evaluation of the Cytotoxic and Antioxidant Activity of *Mentha crispa* Essential Oil, Its Major Constituent Rotundifolone, and Analogues on Human Glioblastoma

**DOI:** 10.1155/2018/2083923

**Published:** 2018-07-02

**Authors:** Hasan Turkez, Ozlem Ozdemir Tozlu, Tamires Cardoso Lima, Anna Emmanuela Medeiros de Brito, Damião Pergentino de Sousa

**Affiliations:** ^1^Department of Molecular Biology and Genetics, Erzurum Technical University, 25200 Erzurum, Turkey; ^2^Department of Pharmacy, University G. d'Annunzio Chieti-Pescara, Via dei Vestini 31, 66100 Chieti, Italy; ^3^Departamento de Farmácia, Universidade Federal de Sergipe, 49100-000 São Cristóvão-SE, Brazil; ^4^Departamento de Ciências Farmacêuticas, Universidade Federal da Paraíba, 58051-970 João Pessoa-PB, Brazil

## Abstract

Cancer is a major public health problem around the globe. This disorder is affected by alterations in multiple physiological processes, and oxidative stress has been etiologically implicated in its pathogenesis. Glioblastoma (GBM) is considered the most common and aggressive brain tumor with poor prognosis despite recent improvements in surgical, radiation, and chemotherapy-based treatment approaches. The purpose of this study was to evaluate antitumor activity from *Mentha crispa* essential oil (MCEO), its major constituent rotundifolone (ROT), and a series of six analogues on the human U87MG glioblastoma cell line. Cytotoxic effects of the compounds on the human U87MG-GBM cell line were assessed using *in vitro* cell viability and oxidative and molecular genetic assays. In addition, biosafety assessment tests were performed on cultured human blood cells. Our findings revealed that MCEO, 1,2-perillaldehyde epoxide (EPER1), and perillaldehyde (PALD) were the most cytotoxic compounds against U87MG cells, with IC50 values of 16.263, 15.087, and 14.888 *μ*g/mL, respectively. Further, these compounds increased the expressions of *BRAF*, *EGFR*, *KRAS*, *NFκB1*, *NFκB1A*, *NFκB2*, *PIK3CA*, *PIK3R*, *PTEN*, and *TP53* genes at different degrees and decreased the expression of some genes such as *AKT1*, *AKT2*, *FOS*, and *RAF1*. Finally, treatment with MCEO, EPER1, and PALD did not lead to genotoxic damage in blood cells. Taken together, our findings reveal antiproliferative potential of MCEO, its major component ROT, and its tested analogues. Some of these chemical analogues may be useful as prototypes for the development of novel chemotherapeutic agents for treating human brain cancer and/or other cancers due to their promising activities as well as nonmutagenic property and safety.

## 1. Introduction

Cancer is a major public health problem, being the second-leading global cause of death after cardiovascular diseases [1, 2]. According to the American Cancer Society, there was an overall estimate of 1,685,210 new cancer cases diagnosed in United States and approximately 600,000 deaths in 2016 [3]. In Europe, over 3 million new cancer cases emerge each year. Glioblastoma (GBM) is the most common and aggressive brain tumor, with a median survival after diagnosis of 12 to 14 months. Despite advances in diagnostic and therapeutic strategies, especially over the last 20 years, GBM continues to show a dismal prognosis [4, 5]. Due to high prevalence and significant morbimortality of this disorder, there is an urgent need to discover new treatment options [6].

Cancer is affected by changes in several physiological processes, including inflammation, apoptosis, oxidant/antioxidant balance, differentiation, and angiogenesis. Oxidative stress, defined as a persistent perturbation between free radical production and antioxidant defenses, has been recognized to be an important contributor to cancer development [7–9]. Studies performed in various types of cancer (brain, prostate, breast, melanoma, colon, and carcinoma) have displayed that oxidative stress players are expressed abnormally in cancers, affecting the phenotype (biological behavior) of cancer cells as well as their ability of response to therapeutic interventions [6, 10, 11].

**Figure 1:**
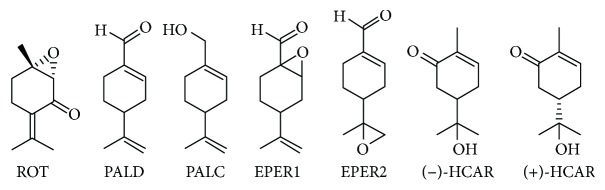
Structures of evaluated compounds.

*Mentha crispa* (syn. Mentha x villosa Hudson), a hybrid species, belongs to the Lamiaceae family. This species is popularly known as “hortelã-da-folha-miúda” or Cuban mint, being extensively cultivated in northeastern Brazil [12]. *M. crispa* essential oil (MCEO) possesses several biological properties, including antinociceptive [13], spasmolytic [14], antimicrobial [15], larvicidal [12], trypanocidal [16], cytotoxic, and antitumoral [17, 18]. Rotundifolone (ROT), a *p*-menthane-type monoterpene, is the major chemical constituent (around 70%) from MCEO [19]. Therefore, the aim of the present study was to evaluate the *in vitro* cytotoxic activity from MCEO, its major constituent ROT, and a series of six related monoterpenes (Figure 1) on the human U87MG-GBM cell line for the first time.

## 2. Material and Methods

### 2.1. Reagents and Chemical Analogues

MCEO was acquired from Hebron company® (Recife, Brazil). Monoterpenoid ROT was isolated from MCEO as earlier described by Almeida and collaborators [19]. MCEO analysis by GC/MS found rotundifolone in 58.11% [16]. This essential oil was submitted to preparative thin-layer chromatography (PTLC) on silica gel and developed with hexane. Plates were exposed under UV light (254 nm), and ROT was visualized as the major component. Afterwards, ROT was removed from chromatographic plates, extracted with CH2Cl2, filtered, and concentrated to give a yellowish oil. Structural characterization of ROT was carried out by infrared, 1H and 13C nuclear magnetic resonance (NMR) analysis, and comparison with literature data [16]. Compound (−)-perillyl alcohol (PALC) (purity 96%, GC) was purchased from Sigma-Aldrich (St. Louis, MO, USA). (+)-Hydroxycarvone [(+)-HCAR], (−)-hydroxycarvone [(−)-HCAR] [20], perillaldehyde (PALD) [21], perillaldehyde 1,2-epoxide (EPER1), and perillaldehyde 8,9-epoxide (EPER2) [22] were prepared according to literature and analyzed by infrared and 1H- and 13C-NMR. Chemical structures of evaluated compounds are shown in Figure 1.

### 2.2. Cell Cultures and Treatments

The human U87MG cell line is extensively used as a model for brain cancer. This cell line was acquired from the American Type Culture Collection (Rockville, MD, USA). The cells were cultured in Eagle's Minimal Essential Medium (EMEM) containing 10% fetal bovine serum (FBS), 1% glutamine, and antibiotics (100 U/mL penicillin and 100 mg/L streptomycin) (Sigma-Aldrich, MO, USA) in an incubator (37°C and 5% CO2, humidified atmosphere). The cells were seeded in 25 mL flasks, and, after reaching to proper volume, they were seeded in 48-cell plates (each well contained 100 mL medium with 1 × 105 cells).

Cells at 70–80% confluence were treated (*n* = 4) with various concentrations (0.78, 1.56, 3.125, 6.25, 12.5, 25, and 50 mg/L) of the MCEO and analogues during 48 h. A stock solution of 10 mg/mL of all compounds and MCEO was prepared in DMSO and diluted to necessary concentration with fresh medium prior to use. Final DMSO concentration in cultures does not exceed 0.1% (*v*/*v*), which did not alter cell growth when compared with controls. The cells grown in media containing DMSO without compounds or essential oil were used as negative control. Triton-X 1%, doxorubicin (DOX), and mitomycin C (10–7 M) were used as positive controls for cell viability and genotoxicity analysis.

### 2.3. In Vitro Evaluation of Cytotoxic Activity by MTT and LDH Assays

#### 2.3.1. MTT Assay

According to the manufacturer's instructions (Cayman Chemical Company®, Ann Arbor, MI, USA), a 3-(4,5-dimethylthiazol-2-yl)-2,5-diphenyltetrazolium bromide (MTT) solution was added to cell cultures and incubated for 3 h. After incubation, DMSO was employed to solve formazan crystals. The cellular viability was determined by measuring the absorbance at 570 nm in a microplate reader. All experiments were performed in quadruplicate. We followed the methods of Cacciatore et al. [23]. The half-maximal inhibitory concentration values (IC_50_) were estimated from MTT assay using probit analysis.

#### 2.3.2. LDH Assay

For LDH assay application, LDH cytotoxicity assay kit (Cayman Chemical Company, Ann Arbor, MI, USA) was employed according to the manufacturer's recommendations. Initially, the cells were moved to 48-well plates and treated with several concentrations of test compounds for 48 h. Then, supernatant (100 *μ*L) and reaction mixture (100 *μ*L) were transferred to a fresh 48-well plate and incubated at room temperature for 30 min. The absorbance was measured in a microplate reader at 490 nm [24].

### 2.4. Total Antioxidant Capacity (TAC) and Total Oxidant Status (TOS) Assays

The levels of total antioxidant capacity (TAC) and total oxidant status (TOS) were estimated on plasma samples of treated and untreated cultures (48 h) employing commercially available kits (Rel Assay Diagnostics®, Turkey) according to the manufacturer's instructions.

### 2.5. Apoptosis Detection by Hoechst 33258 Staining

Hoechst 33258 staining was utilized to visualize nuclear changes and apoptotic body after treatment with selected compounds and MCEO. The cultures were treated with control, compounds, and/or MCEO and incubated during 48 h to analyze cell morphology. The cells were fixed in phosphate-buffered saline at 4°C for 30 min with 4% *p*-formaldehyde. Afterwards, the cells were washed in PBS, and nuclear DNAs were incubated with Hoechst 33258 fluorescent dye (1 M) at room temperature for 5 min. The morphological changes of cell nuclei were observed and photographed under fluorescence microscopy (Leica® DM IL LED).

### 2.6. Total RNA Isolation, cDNA Synthesis, and PCR Array

The cells (5 × 10^6^) were seeded in a 6-well plate, containing 2 mL growth medium, and treated with IC_50_ concentrations from selected compounds and MCEO for 48 h (5% CO_2_). Total RNA was isolated using PureLink® RNA Mini Kit (Invitrogen, MA, USA) according to the manufacturer's instructions. The RNA concentration was evaluated by measuring the absorbance at 260 nm in a microplate reader (Multiskan, Thermo Labsystems, Finland). The synthesis of cDNA from a RNA template, via reverse transcription, was performed using a High-Capacity cDNA Reverse Transcription kit (Applied Biosystems) according to the manufacturer's protocol. Total cDNA was utilized in expression analysis by Custom TaqMan® Assay (Applied Biosystems). Plates were designed with the *18SRNA* housekeeping gene and other 15 genes (*EGFR*, *AKT1*, *AKT2*, *NFKB1*, *NFKB1A*, *NFKB2*, *PTEN*, *KRAS*, *PIK3CA*, *PIK3R1*, *TP53*, *RAF1*, *BRAF*, *DVL1*, and *FOS*) involved in cancer development and progression. RT-PCR assay was done with a PCR system (7500 Fast Real-Time PCR; Applied Biosystems, USA), and PCR reactions (5 *μ*L) were performed with cDNA (30 ng), 2x TaqMan Universal Master Mix buffer (Applied Biosystems), 20x primer, and probe Mix (Applied Biosystems). Thermal cycling conditions included a 20 s denaturation step at 95°C followed by 40 cycles of 3 s at 95°C and 30 s at 62°C. Threshold cycle (CT) was determined and analyzed on an Applied Biosystems 7500 Fast System SDS software. CT values were normalized to an endogenous control and utilized to compare with control samples, giving differential expression profiles.

### 2.7. Biosafety Evaluation

Biosafety from MCEO and selected compounds was evaluated using cultured human peripheral blood cells. Blood cultures were set up according to a slight modification of protocol described by Evans and O′ Riordan [25]. Human blood samples were acquired from four men, aged 26 to 28 years, healthy, nonalcoholics, not under drug therapy, nonsmoking, and with no recent history of exposure to mutagens. 0.8 mL heparinized blood was cultured in 7.0 mL of culture medium (PB-MAX Karyotyping Medium Gibco, Barcelona, Spain) containing 5.0 mg/mL of phytohemagglutinin (Sigma-Aldrich, Steinheim, Germany). Several concentrations from test compounds and MCEO were added to the cultures before incubation. Cytotoxic and genotoxic potentials from test compounds and MCEO were examined by MTT, LDH, sister chromatid exchange (SCE), and 8-hydroxy-2′-deoxyguanosine (8-OH-dG) assays.

### 2.8. SCE Testing

In order to furnish successive visualization of sister chromatid exchanges (SCEs), a 5-bromo-2-deoxyuridine (Sigma-Aldrich) solution was added to culture initiation. Demecolcine (*N*-diacetyl-*N*-methylcolchicine, Sigma-Aldrich) was added to the cultures 70 h and 30 min after the beginning of incubation. The cell suspension was treated with hypotonic solution (0.075 M, KCl), three repetitive cycles of fixation in MeOH/CH3CO2H solution (3 : 1, *v*/*v*), centrifugation, and resuspension. Then, this cell suspension was dropped onto chilled, grease-free microscopic slides, air-dried, aged for three days, and differentially stained for the inspection of SCE rate according to fluorescence plus Giemsa (FPG) procedure. Well-spread thirty-second division metaphases containing 42–46 chromosomes per cell were scored for each treatment condition. Obtained values were estimated as SCEs per cell.

### 2.9. Nucleic Acid Oxidation

8-OH-dG assay kits were purchased from Cayman Chemical for determining 8-OH-dG levels in cultures. All procedures were performed in accordance with the provider's manual [26].

### 2.10. Statistical Analysis

Results were expressed as mean ± SD from at least four independent experiments. For statistical comparisons, quantitative data were analyzed by one-way analysis of variance (ANOVA) followed by Duncan's test using the statistical program SPSS software (version 20.0, SPSS, Chicago, IL, USA). A *p*value < 0.05 was regarded as significant.

## 3. Results and Discussion

### 3.1. Effect of Tested Compounds and MCEO on Cytotoxicity and Apoptosis

Mitochondrial dehydrogenase function by MTT reduction and membrane damage by lactate dehydrogenase (LDH) leakage were assessed as cytotoxicity endpoints. The use of MTT is the most common strategy for revealing antiproliferative actions by natural compounds and/or extracts on several cancer cell lines [27, 28]. However, the MTT test could exhibit false-positive results when examining natural products with intrinsic reductive potential [29]. In fact, various natural products including kaempferol and resveratrol led to increases in MTT-reducing activity *in vitro* [30]. Since the presence of the possibility of underestimation of the antiproliferative potentials by MTT assay depends on the chemical structure of natural compounds [31], we also performed LDH assay apart from MTT analysis. It is well known that only LDH retained its activity through cell viability testing. In this regard, LDH was recently reported as one of the most appropriate assays for establishing the cytotoxicity and/or determining the noncytotoxic concentrations of novel phytotherapeutic agents [32].

**Figure 2:**
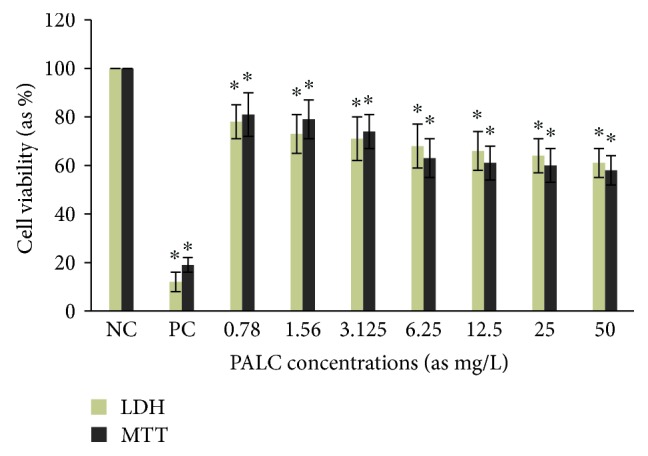
Cytotoxic effect of PALC in U87MG cells for 48 h. Data are presented as mean ± SD of four repetitions. NC: negative control; PC: positive control (Triton-X, 1%). ^∗^*p* < 0.05, compared to NC.

**Figure 3:**
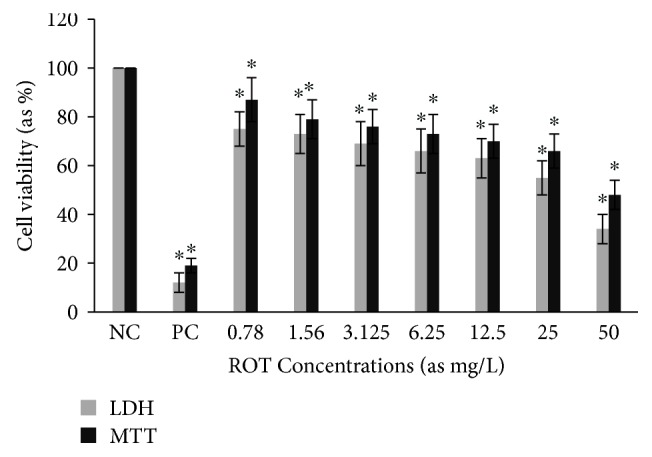
Cytotoxic effect of ROT in U87MG cells for 48 h. Abbreviations are as described in Figure 2. ^∗^*p* < 0.05, compared to NC.

**Figure 4:**
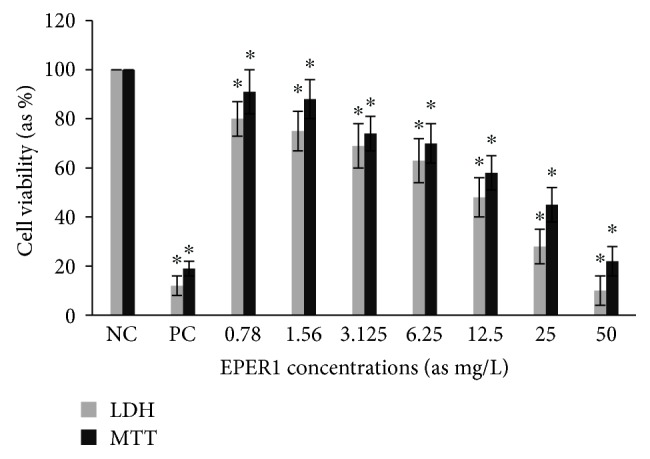
Cytotoxic effect of EPER1 in U87MG cells for 48 h. Abbreviations are as described in Figure 2. ^∗^*p* < 0.05, compared to NC.

**Figure 5:**
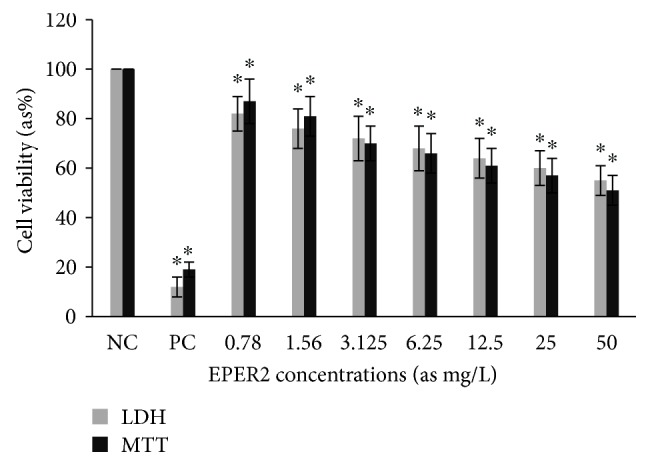
Cytotoxic effect of EPER2 in U87MG cells for 48 h. Abbreviations are as described in Figure 2. ^∗^*p* < 0.05, compared to NC.

**Figure 6:**
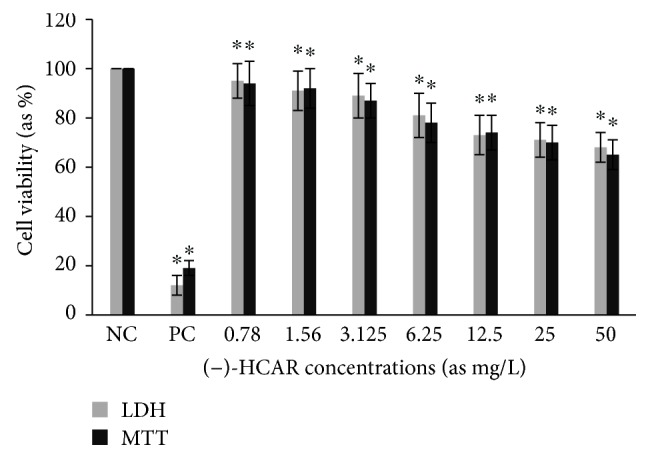
Cytotoxic effect of (−)-HCAR in U87MG cells for 48 h. Abbreviations are as described in Figure 2. ^∗^*p* < 0.05, compared to NC.

**Figure 7:**
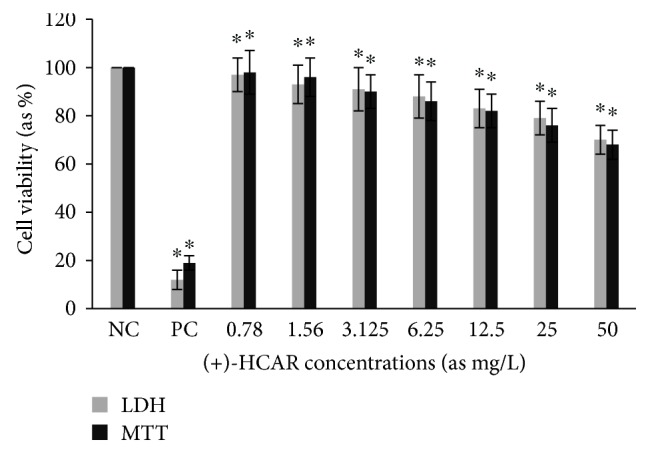
Cytotoxic effect of (+)-HCAR in U87MG cells for 48 h. Abbreviations are as described in Figure 2. ^∗^*p* < 0.05, compared to NC.

**Figure 8:**
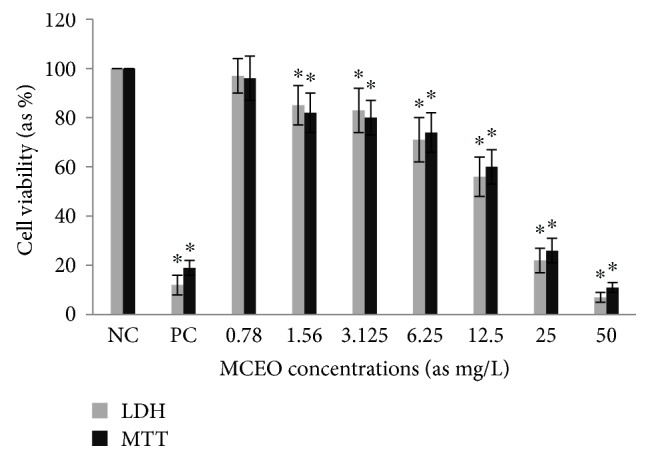
Cytotoxic effect of MCEO in U87MG cells for 48 h. Abbreviations are as described in Figure 2. ^∗^*p* < 0.05, compared to NC.

**Figure 9:**
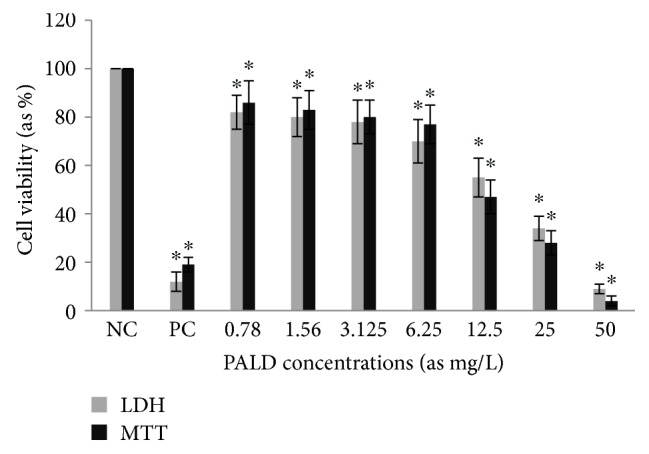
Cytotoxic effect of PALD in U87MG cells for 48 h. Abbreviations are as described in Figure 2. ^∗^*p* < 0.05, compared to NC.

Cytotoxic activity from MCEO, its major constituent ROT, and a series of six related monoterpenes were evaluated against the human U87MG-GBM cell line. Both MTT and LDH analysis revealed a concentration-dependent cytotoxicity after treatment with MCEO, ROT, PALD, EPER1, EPER2, (−)-HCAR, (+)-HCAR, and PALC, and in a concentration range of 0.78–50 mg/L. Percentages of cell viability obtained with continuous exposure for 48 h are depicted in Figures 2–9. Cell viability decreased linearly at increasing concentrations of all tested samples and was expressed as 50% inhibitory concentration (IC_50_). IC_50_ values were calculated via MTT assay (Table 1). When comparing IC50 values from MCEO and seven chemical analogues, MCEO, EPER1, and PALD appeared to be the more active against the proliferation of U87MG cells than other tested compounds did, with IC50 values of 16.263, 15.087, and 14.888 *μ*g/mL, respectively. Therefore, further experiments were performed with these three most effective samples.

**Figure 10:**
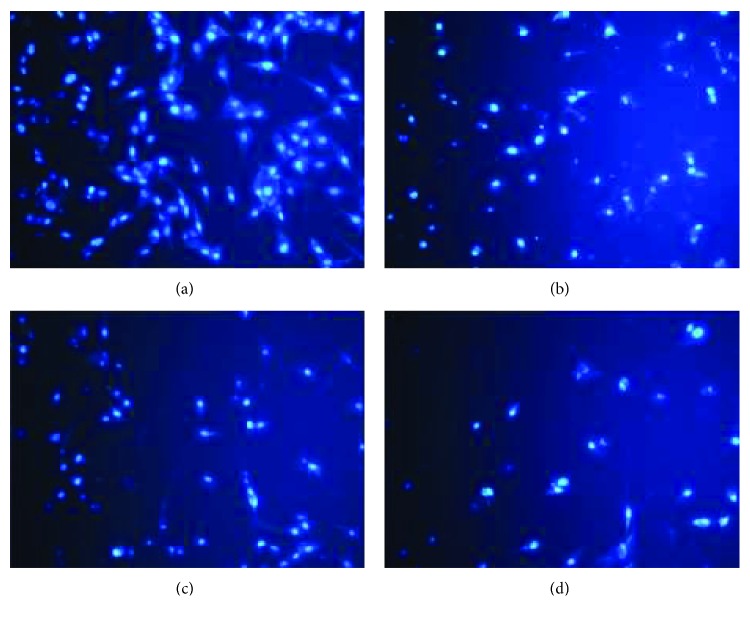
Hoechst 33258 staining in glioblastoma cells. Cells were exposed to various compounds for 48 h. (a) Control, (b) MCEO, (c) PALD, and (d) EPER1.

Cell apoptosis was observed by Hoechst 33258 staining and photographed under a fluorescence microscope (×400 magnification). U87MG cells were treated only with MCEO, EPER1, and PALD during 48 h at their IC50 concentrations. Apoptotic cells, detected by fluorescence microscopy, displayed typical changes of apoptosis, including staining bright of condensed and/or fragmented nucleus. Hoechst apoptosis-detection staining established augmented chromatin condensation and fragmented nucleus in treated cells with MCEO, EPER1, and PALD as compared to untreated cells under fluorescence microscope (Figure 10).

Previous studies have demonstrated that essential oils extracted from aromatic plants as well as their chemical constituents exhibit important biological activities, including effect against different cancer cell lines [33]. There is a relationship between ROS overproduction and origin of oxidation and inflammation, facts which can lead to transformation of normal cells into tumor cells [34–36]. For example, *Cinnamomum cassia* essential and *trans*-cinnamaldehyde, its main component, exhibited potent antimelanogenic activity in murine B16 melanoma cells stimulated with *α*-melanocyte-stimulating hormone (*α*-MSH), and this effect was coupled with antioxidant properties (inhibition of oxidative stress) [37]. Melanogenesis has been reported to involve the production of hydrogen peroxide (H2O2) by means of enzymatic and nonenzymatic reactions as well as subsequent generation of other ROS, causing oxidative stress for melanocytes [38, 39]. Further, *α*-MSH-induced melanogenesis is related with ROS generation [40]. Similarly, other plant-derived natural products (curcumin, gallic acid, vanillin, and ascorbic acid) with antioxidant properties also have demonstrated antimelanogenic effects [41–44].

### 3.2. Antioxidative/Prooxidative Effects of MCEO, EPER1, and PALD on U87MG Cells

**Figure 11:**
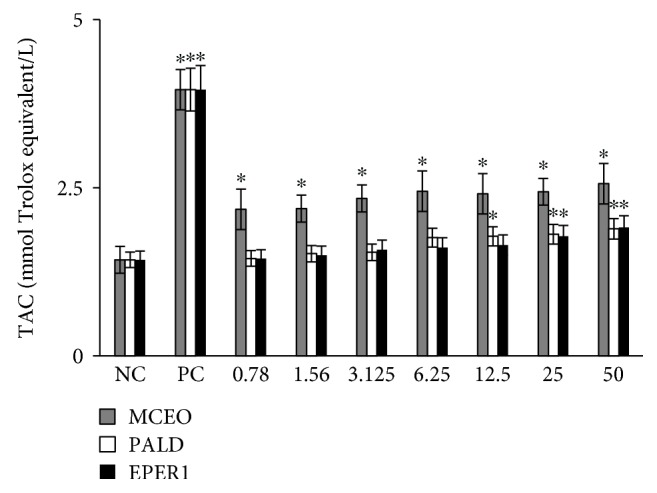
Levels of total antioxidant capacity (TAC) in U87MG cells treated with MCEO, PALD, and EPER1 for 48 h. NC: negative control; PC: positive control (ascorbic acid, 10 *μ*M). ^∗^*p* < 0.05, compared to the NC.

**Figure 12:**
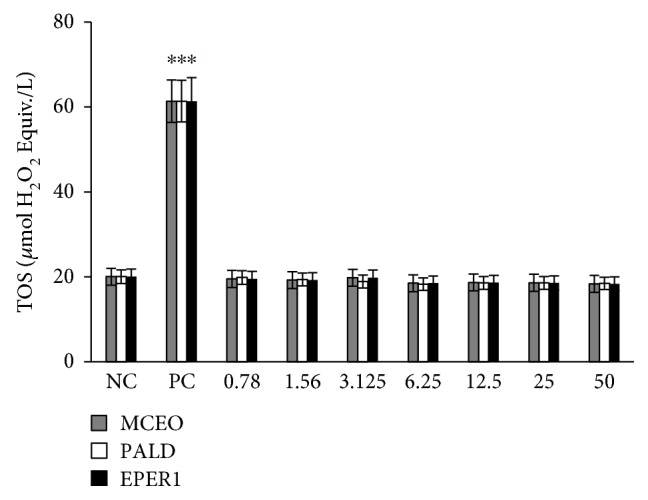
Levels of total oxidant status (TOS) on U87MG cells treated with MCEO, PALD, and EPER1 for 48 h. NC: negative control; PC: positive control (hydrogen peroxide, 25 *μ*M). ^∗^*p* < 0.05, compared to the NC.

Total antioxidant capacity (TAC) and total oxidant status (TOS) levels were evaluated in samples from treated and untreated cultures using an automated colorimetric measurement method. As shown from the results presented in Figure 11, all tested concentrations (from 0.78 to 50 mg/L) from MCEO resulted in increases in TAC levels on U87MG cells compared with the controls. Likewise, relatively higher concentrations of PALD (12, 25, and 50 mg/L) and EPER1 (25 and 50 mg/L) supported antioxidant capacity *in vitro*. On the other hand, treatments with MCEO, EPER1, and PALD did not change the TOS levels in cultured U87MG cells at all applied concentrations (Figure 12).

Similar to our findings, previous reports have indicated that essential oils and their major components could be a potential alternative for glioblastoma treatment. In fact, essential oil from *Hypericum hircinum* exhibited a high antiproliferative activity on the human T98G glioblastoma cell line. Likewise, essential oil from *Zanthoxylum tinguassuiba* showed significant inhibition rates against glioma cells [33]. Harzallah and collaborators [45] investigated the potential protective effect of monoterpene thymoquinone (TQ), the *Nigella sativa* essential oil active compound, in an experimental model of colon cancer induced by procarcinogen 1,2-dimethylhydrazine (DMH). Treatment with TQ significantly elevated the malondialdehyde (MAD) and conjugated diene (CD) content and reduced ROS levels in erythrocytes, attenuating the peroxidation. Further, TQ also provoked an overexpression of the antioxidant defense system, the first line of cellular defense against oxidative damage. These results suggested that TQ protected against oxidative damages caused by DMH in erythrocyte by increasing the activity of antioxidant enzymes (counteracting the oxidative stress) and inhibiting lipid peroxidation [45].

Apart from the major presence of ROT, other minor compounds such as carvone, limonene, pinene, myrcene, and cineol were found in MCEO [46, 47]. These compounds also exhibited weak to moderate antioxidative and antiproliferative properties. In fact, carvone was found to have an antioxidative effect with chelating properties of Fe^2+^, DPPH radical-scavenging activity. Moreover, the higher concentrations (>100 mg/L) of carvone led to significant decreases in cell viability rates in primary rat neuron and N2a neuroblastoma (NB) cell cultures [48, 49]. Likewise, limonene, at relatively high concentrations (125–1800 mg/L), supported the antioxidant capacity against ROS generation after *in vitro* exposure to H_2_O_2_ [50]. The anticarcinogenic properties of limonene were associated with gene expression alterations in apoptosis, signal transduction, DNA damage repair, and cell cycle regulation pathways [51]. Again, both antioxidative and antiproliferative potentials of pinene [52, 53], myrcene [54, 55], and 1,8-cineol [56, 57] were reported by different researchers.

### 3.3. Molecular Responses to MCEO, EPER1, and PALD on U87MG Cells

Gene expression changes induced by treatment with MCEO, EPER1, and PALD on U87MG cells were examined using mini-microarray analysis. MCEO, EPER1, and PALD concentrations (at IC_50_ values) were selected for array hybridization. These samples led to increases in *BRAF*, *EGFR*, *KRAS*, *NFκB1*, *NFκB1A*, *NFκB2*, *PIK3CA*, *PIK3R*, *PTEN*, and *TP53* gene expression at different degrees in the U87MG cell line compared to untreated subjects. Gene *NFκB1A* has been often heterozygously deleted in nonclassical subtypes of GBM patients. Bredel and collaborators [58] showed that deletion of *NFκBIA* (encoding nuclear factor of *κ*-light polypeptide gene enhancer in B-cells inhibitor-*α*), an inhibitor of the EGFR-signaling pathway, promoted tumorigenesis in glioblastoma. Moreover, deletion of *NFκBIA* had an effect similar to *EGFR* amplification in pathogenesis of glioblastoma and was associated with comparatively short survival. PTEN (phosphatase and tensin homologue deleted in chromosome 10) tumor-suppressor protein, an inhibitor of the *PI3K* signaling pathway, was commonly lost in glioblastoma [59, 60]. In study performed by Gallia and collaborators [61], it was reported that 15% of glioblastomas possessed *PIK3CA* mutations and these mutations were prevalent in glioblastomas from both pediatric and adult patients. Further, loss of function in tumor suppressors, such as *NF1*, *VHL*, and *PIK3R1*, was reported in glioma [62]. The *TP53* tumor suppressor gene, a transcription factor for numerous genes involved in cell cycle control, DNA repair, apoptosis, and angiogenesis, was one of the most frequently mutated genes in human cancer [63].

**Figure 13:**
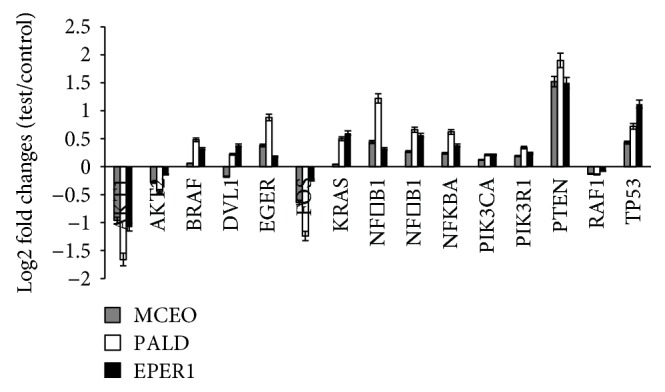
Gene expression patterns after MCEO, PALD, and EPER1 treatments on U87MG cells for 48 h.

We also revealed that expression of genes including *AKT1*, *AKT2*, *FOS*, and *RAF1* were decreased by MCEO, EPER1, and PALD. In addition, the expression of the *DVL1* gene increased after PALD and EPER1 treatment, but decreased after MCEO application (Figure 13). In accordance with our findings, previous studies determined that *AKT1* activity has been associated with glioblastoma invasiveness, a central characteristic behind its lethality [64]. Furthermore, *AKT2* played a critical role in development of gliomas and presented a potential therapeutic target for malignant gliomas [65]. In fact, recent reports have shown that *AKT1* and *AKT2* expression is associated with more advanced and particularly aggressive gliomas [66]. *RAF* signaling played an important role in gliomagenesis, despite apparent absence of genetic abnormalities in *BRAF* and *RAF-1* genes [67]. Using microarrays and quantitative RT-PCR (qRT-PCR), they found increased *FOS* in high-grade gliomas. *FOS* depletion (via FOS-shRNA) inhibited invasion and promoted apoptosis in glioma cells, and abrogating the expression of *FOS* has suppressed the proliferation and invasion and delayed the cell cycle at the G1 phase for both U87 and U251 cells [68, 69].

On the other hand, the epidermal growth factor receptor (*EGFR*) was frequently amplified, overexpressed, or mutated in glioblastoma [59]. *NFκB1* was overexpressed in a high proportion of oral cancer cases [70]. Inhibition of *KRAS* expression resulted in apoptotic tumor regression and increased survival of tumor-bearing mice, strongly suggesting that *KRAS* signaling is required for tumor maintenance *in vivo* [71]. It also seemed that the entire *BRAF* gene was duplicated in some tumors, possibly leading to its overexpression. Low-level genomic gains including *BRAF*, most of which involved all of chromosome 7, have been documented in 17 of 23 World Health Organization grade II to IV gliomas, with 13 of these occurring in glioblastoma [72]. Overexpression of *DVL* has been shown to potentiate the activation of *Wnt* signaling, and it is now apparent that upregulation of *DVL*s is involved in several types of cancer [73].

### 3.4. Biosafety Assessments

#### 3.4.1. Cytotoxicity Testing

**Figure 14:**
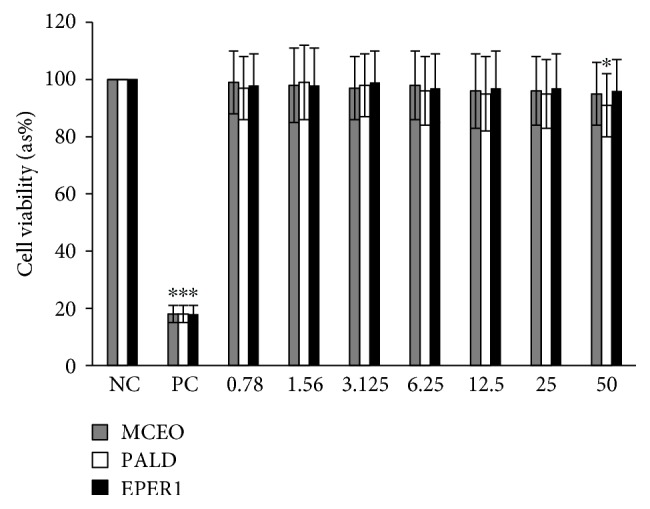
Effects of MCEO, PALD, and EPER1 on the cell viability (MTT assay) of cultured human blood cells. Data are presented as the mean ± SD of four repetitions. NC: negative control; PC: positive control (Triton X 1%). ^∗^*p* < 0.05, compared to NC.

**Figure 15:**
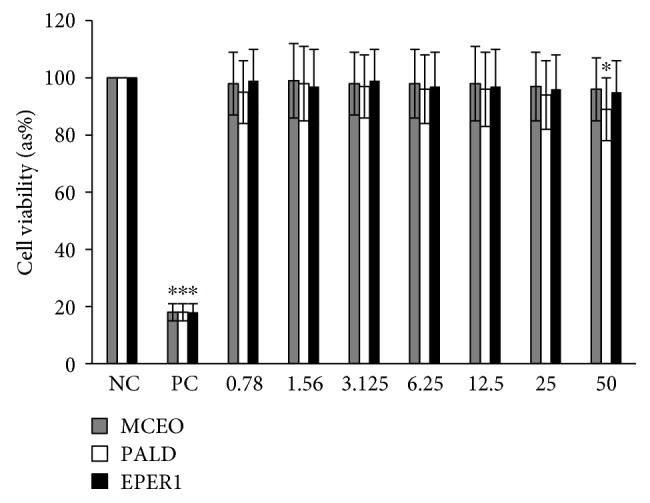
Effects of MCEO, PALD, and EPER1 on the cell viability (LDH assay) of cultured human blood cells. Data are presented as the mean ± SD of four repetitions. NC: negative control; PC: positive control (Triton X 1%). ^∗^*p* < 0.05, compared to NC.

For measuring cell death in response to different concentrations from MCEO, EPER1, and PALD, we performed MTT and LDH assays. Cultured peripheral human whole blood (PHWB) cells were exposed to 0.78 to 50 mg/L of MCEO, EPER1, and PALD. MCEO and compounds tested in all concentrations (except PALD 50 mg/L) did not show any significant (*p* > 0.05) changes in cell viability during 48 h, as determined by MTT and LDH assays (Figures 14 and 15).

#### 3.4.2. Genotoxicity Testing

**Figure 16:**
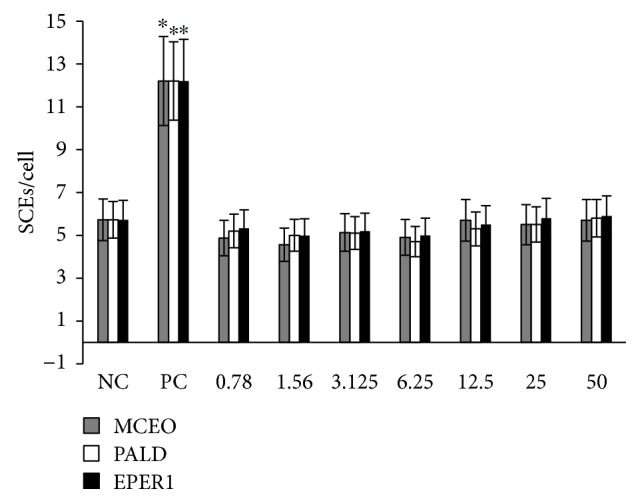
SCE frequencies after treatment with MCEO, PALD, and EPER1 in human lymphocytes. NC: negative control, PC: positive control (mitomycin C, 10^−7^ M). ^∗^*p* < 0.05, compared to NC.

**Figure 17:**
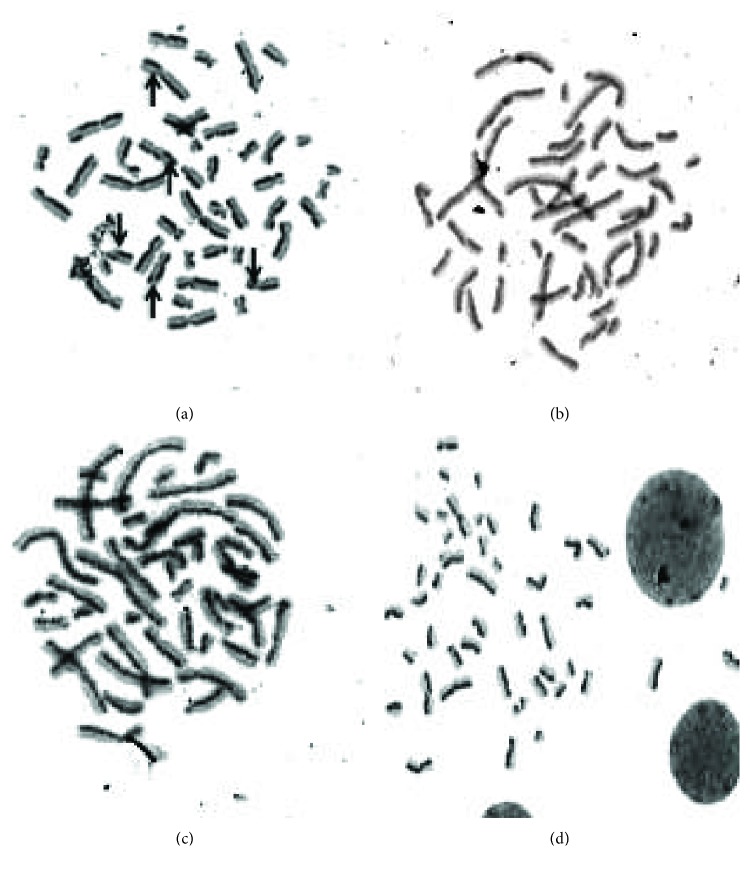
Sample metaphase micrographs from (a) positive control, MMC, and (b) MCEO- (50 mg/L), (c) PALD- (50 mg/L), and (d) EPER1- (50 mg/L) treated cultures (arrows show SCE formations).

**Figure 18:**
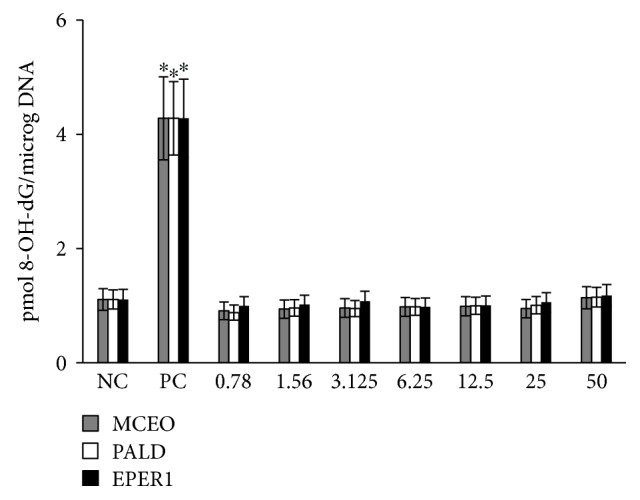
The levels of 8-OH-dG in cultured human blood cells after treatment with MCEO, PALD, and EPER1 for 72 h. NC: negative control; PC: positive control (mitomycin C, 10^−7^ M). ^∗^*p* < 0.05, compared to NC.

To assess whether genotoxic damage increases with increasing MCEO, EPER1, and PALD concentrations, SCE formations were scored in cultured primary human lymphocytes. As presented in Figure 16, there were no significant differences in observed rates of SCEs between the control group and the MCEO-, EPER1-, and PALD-treated groups (*p* > 0.05) (Figure 17). The status of 8-OH-dG in human whole blood cell cultures after treatment with MCEO, EPER1, and PALD is reflected in Figure 18. It was observed that MMC (at 10^−7^ M) significantly increased 8-OH-dG concentrations in cultured human blood cells after 72 h. Contrariwise, 8-OH-dG levels were not changed when treated with EPER1, MCEO, and PALD concentrations. In brief, after performed cytotoxicity (MTT and LDH assays) and genotoxicity (SCE test and 8-OH-dG level) analysis, treatments with MCEO, EPER1, and PALD were revealed to be safe and biocompatible towards human blood cells.

## 4. Conclusions

In conclusion, the present findings revealed antioxidant and antiproliferative activities of MCEO, ROT, and chemical analogues against the human U87MG-GBM cell line. Observed cytotoxicity results determined that MCEO, EPER1, and PALD exhibited stronger cytotoxic action. The molecular genetic response studies indicated that mainly the alteration of *PTEN/PI3K/AKT/NFκB* signaling pathways played a key function underlying the molecular mechanism occurring due to MCEO, EPER1, and PALD treatments. Thus, these compounds may be used in the investigation of novel chemopreventive or chemotherapeutic agents for human brain and other cancers because of their promising activities and nonmutagenic and safe properties and may be considered for further clinical studies in drug development.

## Figures and Tables

**Table 1:** The IC_50_ values of *Mentha crispa* essential oil and test compounds against U87MG cells.

Compounds	IC_50_ (mg/L)
PALC	78.376 ± 0.63
ROT	30.083 ± 0.81
EPER1	15.087 ± 0,58
EPER2	59.986 ± 0.79
(−)-HCAR	31.243 ± 0.66
(+)-HCAR	72.311 ± 0.85
MCEO	16.263 ± 0.58
PALD	14.888 ± 0.60
DOX	4.020 ± 0.32
Triton-X	8.186 ± 0.55

Data are presented as mean ± SD of four repetitions. Triton-X and doxorubicin (DOX) were used as positive controls.

## Data Availability

The data used to support the findings of this study are available from the corresponding author upon request.
